# Comparative evaluation of incorporation calcium silicate and calcium phosphate nanoparticles on biomimetic dentin remineralization and bioactivity in an etch-and-rinse adhesive system

**DOI:** 10.4317/jced.59817

**Published:** 2022-11-01

**Authors:** Zohre Ashtijoo, Leila Pishevar, Mohammad-Reza MalekipourMalekipour, Mohammad Khodaei, Zahra Sabouri

**Affiliations:** 1 Private Dentist, Department of Operative Dentistry, Faculty of Dentistry, Isfahan (Khorasgan) branch, Islamic Azad University, Isfahan, Iran; 2 Assistant Professor, Department of Operative Dentistry, Faculty of Dentistry, Isfahan (Khorasgan) branch, Islamic Azad University, Isfahan, Iran; 3 Associated Professor, Department of Operative Dentistry, Faculty of Dentistry, Isfahan (Khorasgan) branch, Islamic Azad University, Isfahan, Iran; 4 Materials Engineering Group, Golpayegan College of Engineering, Isfahan University of Technology, Golpayegan, 87717-67498, IranMaterials Engineering Group, Golpayegan College of Engineering, Isfahan University of Technology, Golpayegan, 87717-67498, Iran; 5 Department of Materials Engineering, Isfahan University of Technology, Isfahan84156-83111, Iran

## Abstract

**Background:**

This study aimed to evaluate the remineralization potential and bioactivity of adhesives, containing amorphous calcium phosphate (ACP) and calcium silicate (CS) nanoparticles (NPs).

**Material and Methods:**

In this study, dentin slices (n=60) were prepared and etched with phosphoric acid. Next, they were divided into two groups: pre- and post-immersion in a simulated body fluid (SBF) for three weeks. The two groups were also divided into five subgroups (n=6 per subgroup), including the control (0 wt.% NPs); adhesives containing 1 wt.% and 2.5 wt.% (CS) nanoparticles; and adhesives containing 1 wt.% and 2.5 wt.% ACP nanoparticles. The remineralization potential and bioactivity of the adhesives were evaluated. The shear bond strength of the samples (n=18) was also assessed using a universal testing machine.

**Results:**

The present results revealed that the adhesive containing ACP and CS nanoparticles showed bioactivity and remineralization potential without any reduction in the bond strength.

**Conclusions:**

The outcomes revealed that Cs and ACP nanoparticles induced mineralization in the dentin and incorporation of these nanoparticles to dentin bonding agents could improve the bio-functionalization of dentin bond.

** Key words:**Calcium phosphate, calcium silicate, fourier transform infrared spectroscopy, scanning electron microscopy, tooth remineralization.

## Introduction

In dental caries, acids cause demineralization by affecting the hydroxyapatite crystals and in the organic dentin matrix (1). On the other hand, in the remineralization process, the remaining apatite crystals grow in the dentin (2). Precipitation of minerals, such as calcium and phosphate ions, from materials containing calcium phosphate fillers, besides reaching saturation for apatite deposition, may facilitate the growth of apatite crystals with a remineralized texture, which is probably more resistant to degradation and can effectively repair dental damage (3).

NPs, such as bioactive glass, hydroxyapatite, casein phosphopeptide (CPP), and amorphous calcium phosphate (ACP), have been added to dental adhesives, showing favorable ionic changes and mineral deposition within the hybrid layer (4). NPs can penetrate into the dentin structure due to their very small size (5). However, previous studies have shown that the addition of fillers can increase the bonding agents viscosity and subsequently reduce the dentin surface wetting and the adhesive penetration into the dentin (6).

Calcium phosphate-based biomaterials are important components in hard tissue regeneration due to their high bioactivity and biocompatibility (7). ACP has been considered as a new factor in preventing dental caries (8). A higher release of ions has been shown in ACP NPs compared to other calcium phosphate phases because of their amorphous structure, low crystallinity, and high surface activity, which increase the rates of solubility and resorption, as well as bioactivity (9,10). Besides, calcium silicate (CS) is widely used owing to its high moisture tolerance, biocompatibility, and bioactivity. By releasing calcium and hydroxyl ions, these substances alkalize the environment and provide suiTable conditions for the apatite formation (11,12). Besides, CS is an active and soluble calcium phosphate, which leads to the transformation of calcium and phosphate ions to apatite and helps restore demineralized areas (12).

In this regard, Sadat-Shojaei *et al*. synthesized hyaluronic acid (HA)-NP filler-incorporated adhesives to enhance their mechanical properties (5). Moreover, Yang *et al*. produced silica NP fillers and compared them with some commercial products and fillers (13). Besides, Profeta *et al*. fabricated adhesives doped with bioactive CS-based micro-fillers to generate resin-dentin interfaces (4). Liang *et al*. investigated dental remineralization using poly(amidoamine) polymer materials, containing calcium phosphate NPs (14). Additionally, Prati *et al*. fabricated CS cements for clinical applications and reported the improvement of mechanical and physical properties of these materials (15).

Moreover, Solhi *et al*. modified spherical and hybrid NPs in fillers for dentin bonding systems (16). Cao *et al*. also examined the potential of CPP-ACP to induce apatite formation in dentin collagen fibrils (17). In the present study, we examined the effects of CS- and ACP-reinforced adhesion systems regarding their remineralization potential and bioactivity and compared the results with previous studies. To the best of our knowledge, this is the first study to incorporate two types of CS and ACP NPs into adhesives and examine their dental applications.

As mentioned earlier, this study primarily aimed to compare the remineralization potential and bioactivity of different adhesives and to examine their effects on the formation of apatite or mineral complexes on demineralized dentin. Moreover, the number of trace elements on the dentin surface, along with the bond strength, was measured after incorporating the NPs. The null hypotheses to be tested in this study were as follows: (i) There is no significant difference in the bioactivity or remineralization potential of test materials; and (ii) bond strength is not affected by adding NPs.

## Material and Methods

This study was approved by the medical ethics committee of Islamic Azad University of Isfahan, Iran (IR.IAU.KHUISF.REC.1398.244).

-Preparation of CS NPs:

The CS NPs were synthesized by the sol-gel method. Initially, acid hydrolysis with nitric acid (HNO3, Merck, Germany) as a catalyst was used. Next, tetraethyl orthosilicate (TEOS, purity=99.999%, Sigma) was added to the solution as a silica precursor and stirred for 30 minutes. Subsequently, calcium nitrate tetrahydrate (Ca (NO3)2.4H2O; Merck, Germany) was added to the solution and stirred vigorously for one hour. The solution was centrifuged for eight minutes at 6000 rpm until a precipitate was produced. Deionized water was then removed by washing the precipitate with ethanol twice; the obtained precipitate was dried in an oven. Finally, calcination at optimum temperature (650°C, 5h, 3°C.min-1) was done to remove any remnant organics and collect CS NPs (18,19).

-Preparation of ACP NPs:

The ACP NPs were synthesized via a re-precipitation route, which included calcium and phosphate ions. The HA NPs, as the primary suspension in deionized water (2 wt%), were then prepared. Hydrochloric acid (HCl) was added to HA solution to dissolve. Sodium hydroxide (2 M) was immediately added dropwise to form ACP NPs. The ultimate pH (8, 9, 10, and 11) of the synthesis process was controlled by diluted ammonium hydroxide (NH4OH). Finally, the precipitates of ACP NPs were separated by centrifugation, washed with deionized water for 30-40 minutes, and dried in a vacuum oven at 80°C for one hour (9).

-Adhesive preparation:

Commercial ethanol-based one-bottle dentin adhesives (Tetric® N-Bond, Ivoclar Vivadent, Liechtenstein) were prepared in this study. The properties of the formulated adhesive mixtures are presented in Table 1. The as-synthesized ACP and CS NPs were incorporated into the adhesive solution at weight percentages of 0-2.5 wt.% and then homogenized by ultrasonication for 30 minutes. Finally, adhesive mixtures, containing 0 wt.%, 1 wt.%, and 2.5 wt.% ACP and CS NPs, were prepared.

**Table 1:**
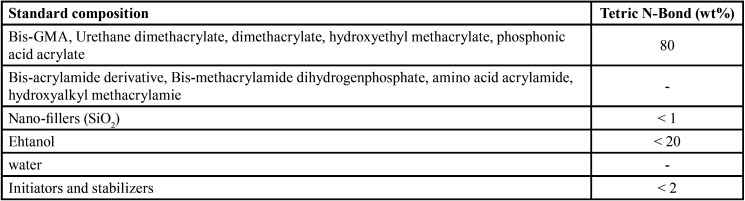
Tetric N-Bond composition.

-Dentin bonding procedures:

Dentin samples were collected from healthy human third molars. The teeth were kept in 0.5% thymol at 4°C for a maximum duration of two months. Dentin slices with a thickness of 1±0.1 mm were prepared, using an automatic cutting machine (CNC automatic cutting machine, Tabriz, Iran). The sections were placed parallel to the cemento-enamel junction (CEJ) of the tooth, using a new diamond-cutting disk with a cooling agent. The occlusal dentin surface was polished with a 600-Grit silicon carbide paper under continuous water irrigation for 60 seconds to produce a standardized smear layer.

The specimens (n=60) were divided into two groups: before (n=30) and after (n=30) immersion. The dentin slices in each group were also randomly divided into five experimental subgroups and treated with the adhesive systems. For this purpose, the exposed flat occlusal dentin surface was etched with etching gel for 15 seconds, rinsed with water, and blot-dried to avoid over-drying of the dentin surface. Two layers of adhesive (0 wt.% (control group), 1 wt.%, and 2.5 wt.% CS and 1 wt.% and 2.5 wt.% ACP) were applied, and the solvent was gently evaporated under a low-pressure air stream until a homogeneous shiny layer was observed on the surface. The adhesive layer was cured with a light-curing unit (intensity, 3200 mw/cm2; Valo, Ultradent, USA) for ten seconds.

-Scanning electron microscopy (SEM)-energy dispersive X-ray (EDX) analysis:

To observe the remineralization of dentin specimens, a SEM analysis was performed. Images were acquired using a Philips instrument (XL30, Netherlands). All SEM samples were gold-coated before observation. The elemental compositions of the samples were also observed using EDX, coupled with SEM.

-Bioactivity test:

The ability to form hydroxyapatite (bioactivity) was assessed by evaluating the formation of apatite on the disc in the presence of simulated body fluid (SBF). In this study, SBF was used to examine bioactivity. For this purpose, a Teflon generator was placed on a transparent matrix strip, which was placed on a glass slab. Next, three drops of each prepared band were injected using a dropper. Also, a pure band was injected into the generator. The discs were then cured with a light-curing unit. The samples removed from the generator were each 5 mm in diameter and 2 mm in thickness. Each glass contained disk samples soaked in 15 mL of SBF, which were incubated at 37°C for three weeks. Finally, the surface morphology and chemistry (surface composition and elemental distribution) were evaluated using SEM-EDX, X-ray diffraction analysis (XRD), and attenuated total reflectance (ATR)-Fourier transform infrared spectroscopy (FTIR) analyses.

-FTIR-ATR analysis:

An FTIR spectrometer (Tensor 27, Bruker, USA), equipped with an ATR system and a deuterated triglycine sulfate (DTGS) detector, was used to determine the chemical structure of the samples. The spectral resolution was 4 cm-1 and 64 cm-1 the number of scan for each spectrum in the region of 651-4111 cm-1; the disk diameter was 2 mm; and the penetrating power was about 2 μ. Due to the high infrared (IR) absorption of water, spectroscopy was performed on the desiccant dried samples. To reduce the problems caused by sample heterogeneity, five IR spectra were collected from the surface of each sample.

-XRD analysis:

The XRD patterns were determined using a Philips X’PERT MPD system with CuKα radiation (λ=1.4506 nm) between 2θ angles of 10 ° and 80 ° at room temperature (40 kV and 32 mA).

-Shear bond strength test:

To measure the shear bond strength, a total of 18 dentin discs were randomly divided into three groups and prepared as previously described, that is, they were impregnated with the prepared bands. Next, a silicone mold (internal diameter, 5 mm; height, 2 mm) was placed on the dentin surface and filled incrementally with the resin composite (IPS Empress, Ivoclar Vivadent AG, Liechtenstein), using the layering technique. Each layer with a maximum thickness of 1 mm was cured for 40 seconds with a Valo light-curing device at an intensity of 3200 nm/cm2. The samples were kept in distilled water at room temperature for 24 hours and then mounted on an acrylic generator, suiTable for a bond strength test machine; the teeth were placed in a way that their bonded surface was parallel to the generator base. The shear bond strength of the samples was measured in a universal testing machine (Walter+Bai AG, Löhningen, Switzerland) at a speed of 1 mm/min. Finally, the shear bond strength was reported in MPa.

-Statistical analysis:

The results were analyzed in IBM SPSS Version 25. Qualitative variables are presented as frequency and percentage, and quantitative variables are presented as mean and standard deviation (SD). Also, analysis of variance (ANOVA) test was used to confirm the bond strength.

## Results

-Apatite formation potential

-SEM l: -EDX analysis.

**Figure 1:**
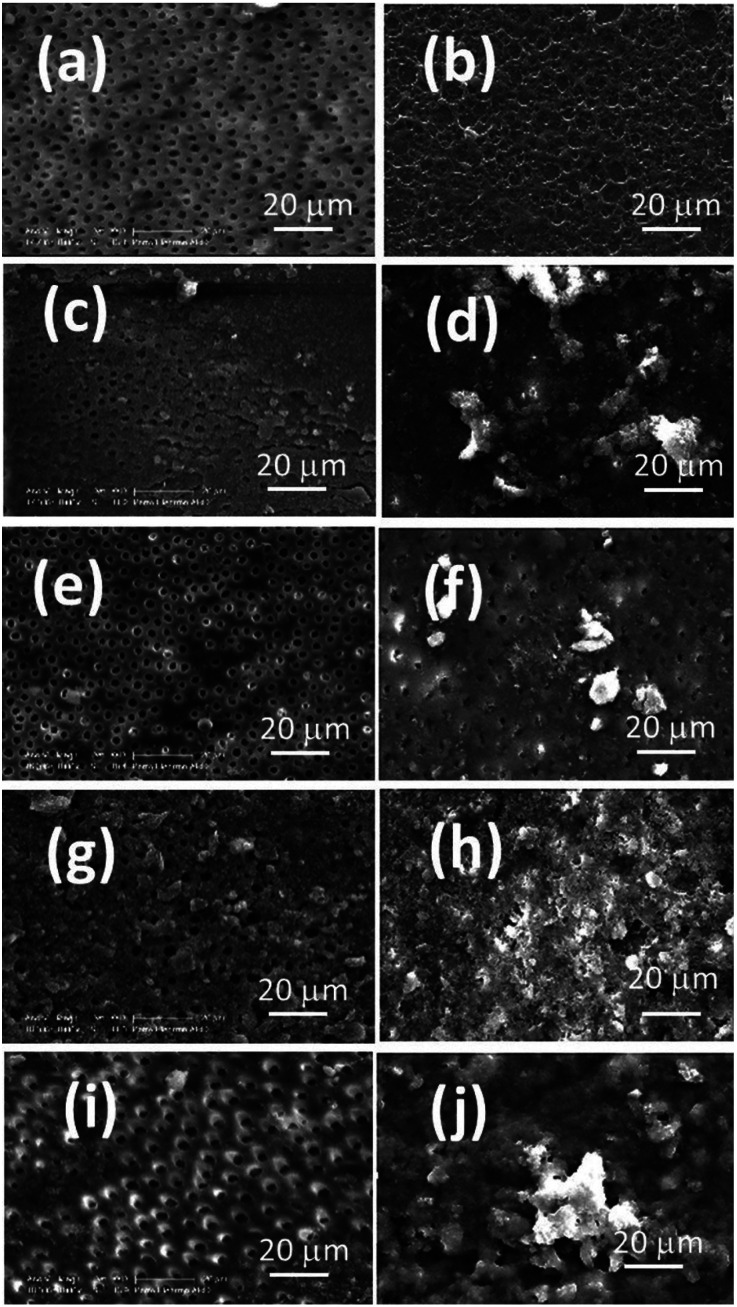
The SEM images of samples. a) Control Group before Immersion, b) control group after immersion, c) calcium silicate 1% before immersion, d) calcium silicate 1% after immersion, e) calcium silicate 2.5% before immersion, f) calcium silicate 2.5% after immersion, g) ACP 1% before Immersion, h) ACP 1% after Immersion, i) ACP 2.5% before Immersion, j) ACP 2.5% after Immersion.

The remineralization process was evaluated following the immersion of dentin disc, reinforced with pure bond and bonds containing different concentrations of CS or ACP NPs in SBF for 21 days, using the SEM-EDS analysis. Figure 1 presents the SEM images of the surfaces of dentin samples after immersion in SBF, covered by some depositions that may be hydroxyapatite. The formation of hydroxyapatite on the surface of samples was due to its bioactivity.

**Figure 2:**
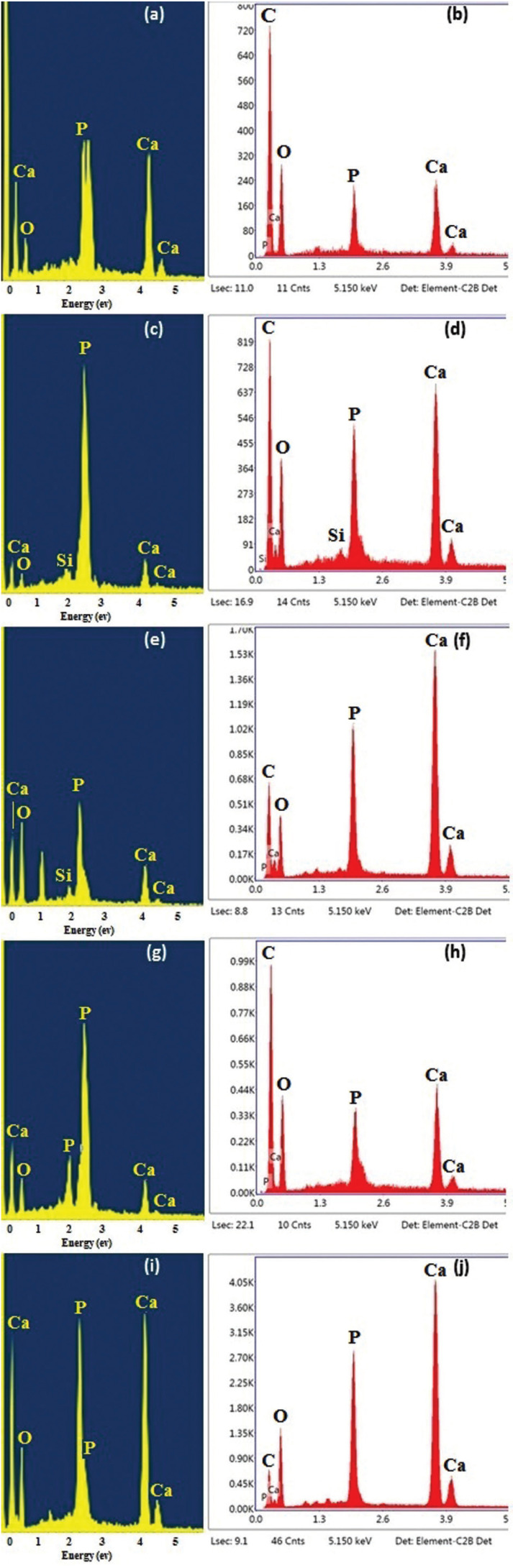
The EDX spectra of samples. a) Control Group before Immersion, b) control group after immersion, c) calcium silicate 1% before immersion, d) calcium silicate 1% after immersion, e) calcium silicate 2.5% before immersion, f) calcium silicate 2.5% after immersion, g) ACP 1% before Immersion, h) ACP 1% after Immersion, i) ACP 2.5% before Immersion, j) ACP 2.5% after Immersion.

To ensure hydroxyapatite formation, an EDS analysis was carried out. The results of EDS revealed an increase in the calcium and phosphorous levels on the surface after immersion in SBF. Since calcium and phosphorous are the major components of hydroxyapatite (Ca10(PO4)6OH2), their presence on the surface may indicate the apatite formation (Fig. 2, Table 2). The control group (without any NPs) showed fewer morphological changes due to immersion in SBF, revealing its lack of bioactivity compared to the other groups. Besides, more apatite was deposited on samples with more NPs. Comparison of Figures 1f, 2f and Figures 1j, 2j indicated that both CS and ACP-reinforced bonds (2.5 wt.%) almost had the same apatite formation potential after immersion in SBF.

**Table 2:**
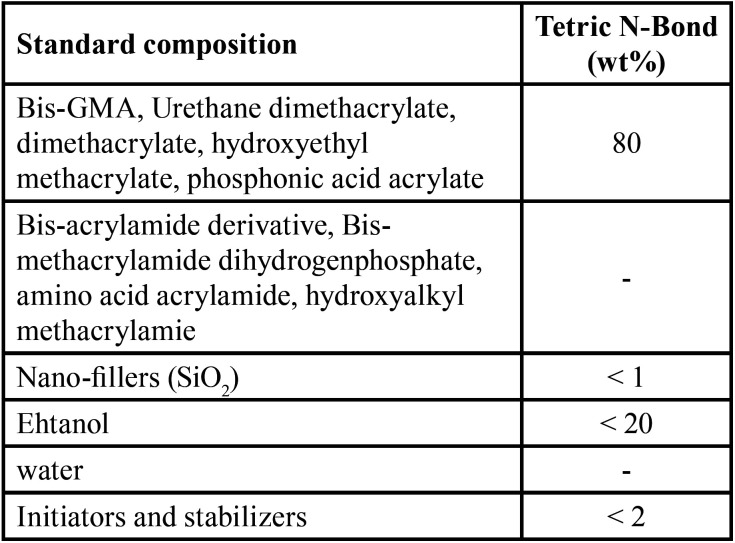
The Calcium and phosphor wt % and Atomic% on test material disk separated by time analyzed by EDX.

-FTIR spectroscopy.

**Figure 3:**
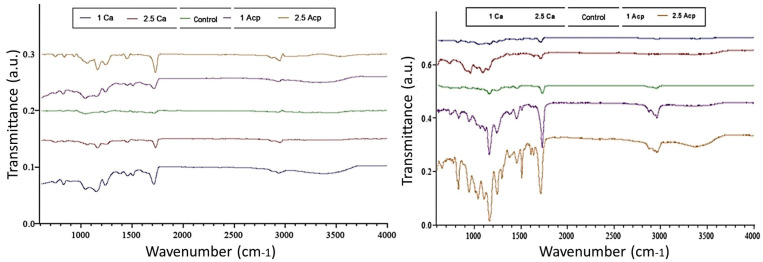
FT-IR spectra of ACP and CS and control group. (a) samples before immersion, (b) after immersion in SBF solution.

Figure 3 presents the FTIR spectra recorded for the surface of the samples (0 wt.% (control group), 1 wt.%, and 2.5 wt.% CS and 1 wt.% and 2.5 wt.% ACP) before and after immersion in SBF. According to Figure 2a, before immersion in SBF, the peaks at 550 and 1000 cm-1 confirm the amorphous property of ACP NPs in bonding. Also, the peaks around 500 and 880 cm-1 were attributed to the presence of Si-O-Ca bands of CS NPs in bonding. In Figure 2b, the spectrum corresponds to the immersed samples in SBF. Phosphate peaks can be seen in ranges of 1000-1100 cm-1 and 947 cm-1, respectively. These peaks represent the formation of an amorphous layer (Ca10(PO4)6OH2) near the surface (20).

Unlike the control group, which showed no significant difference before and after immersion in SBF (no apatite formation on the surface after immersion in SBF), in groups receiving 1 and 2.5 wt.% ACP NPs, the relative intensity of the phosphate peak increased by increasing the time of immersion in SBF. Except for the control group, in other bonding groups, peaks related to carboxylate salts were observed at 1411 and 1511 cm-1, suggesting a small C=O bond (indicative of bioactivity and apatite layer formation). The changes in samples containing 2.5 wt.% ACP and 2.5 wt.% CS were more than the samples containing 1 wt.% CS or ACP. The intensity of peaks formed in the two groups of samples containing different concentrations of ACP was higher than that of samples containing 1 wt.% and 2.5 wt.% CS. In the CS group, the intensity of Si-O peaks decreased over time, indicating hydrolysis of the bioactive glass structure over time.

XRD analysis.

**Figure 4:**
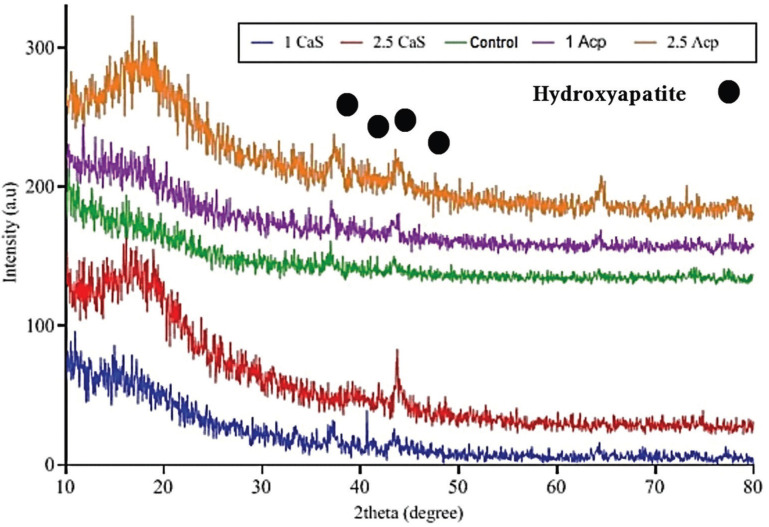
XRD pattern of ACP, CS and control group after 21 days immersion in SBF solution.

Figure 4 presents the XRD diagrams for the control and other groups, containing 1 wt.% and 2.5 wt.% CS and ACP NPs after immersion in SBF for 21 days. The XRD patterns of the samples immersed in SBF present a wide (hill-shaped) peak at the beginning of the pattern, which verifies an amorphous phase in the material. This peak can be seen in almost all samples, although it is more evident in those containing 2.5 wt.% ACP and CS NPs. Sharp and large peaks at positions 33, 36, 39, 43, and 64° corresponded to HA, according to the International Centre for Diffraction Data (ICDD) 9-432 standard (5). These peaks were sharper and larger in the sample containing 2.5 wt.% ACP and CS NPs; therefore, more HA is formed on the surface of this sample. Wide peaks of HA are related to the nanometric scale of hydroxyapatite on the surface.

Figure 4 presents the XRD diagrams for the control sample and other samples containing 1 wt.% and 2.5 wt.% CS and ACP NPs, immersed in SBF solution after 21 days. The ACP NPs changed to a semi-crystalline structure, as the HA growth phase was observed in the structure(19). In samples containing CS NPs, calcium ions from cation exchange with hydrogen ions of the coating, silica-rich gel-like layer on the NP surface, and oversaturated calcium were removed from the coating (21).

-Bond strength:

The results of ANOVA test showed no significant difference in the bond strength of the groups (2.5 wt.% ACP and CS NPs) (*P*=0.414) (Table 3). The addition of ACP or CS NPs up to 2.5 wt.% to the bond had no significant negative effects on the bond strength to dentin.

**Table 3:**
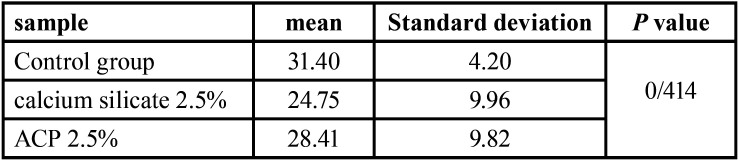
Shear bond strength of the adhesive containing 2.5% ACP, 2.5% CS and control group.

## Discussion

The present study revealed that the addition of CS and ACP NPs to dentin could induce remineralization. According to the results of SEM-EDS analysis, all five groups showed an increase in calcium and phosphate peaks after 21 days of immersion in SBF, indicating hydroxyapatite deposition. As reported by researchers, ACP induces the formation and growth of phosphorylated dentin collagen nuclei within and between fibrils and triggers dentin mineralization through apatite formation(17). As reported by researchers, ACP induces the formation and growth of phosphorylated dentin collagen nuclei within and between fibrils and triggers dentin mineralization through apatite formation. The amount of calcium and phosphate was lower in the control group compared to the other groups. Slight changes in the amount of calcium and phosphate in the control group could be related to the constituents of the body environment simulator and phosphate absorption from the storage solution. Therefore, the first hypothesis of this study (i.e., there is no difference in the remineralization potential between the CS and ACP NP bonds and the control group) was rejected.

The formation of hydroxyapatite can be accelerated by the release of calcium from CS and ACP NPs (in the solution). Calcium ions react with the phosphate groups of SBF and precipitate as calcium phosphate; therefore, hydroxyapatite is nucleated and crystallized on the bonding surface. The hydroxyapatite layer contributes to the physical blockage of dentin tubules. It seems that the prevention of further demineralization can improve the hybrid layer remineralization within the resin-dentin interface in bonding processes.

Rio *et al*. showed that dentin collagen remineralization is possible at the dentin-resin interface via biomimetic remineralization; this was achieved through the gradual release of calcium ions from the Portland cement (tricalcium silicate). They also reported subsequent interactions with phosphate in solid bleached sulfate (SBS) or the substrate (22).

Moreover, Daneshpour *et al*. quantitatively compared the presence of calcium and phosphorus peaks on demineralized dentin due to biomimetic remineralization of commercial bioactive materials (Theracal, Biodentine, and CPP-ACP) and also examined bioactivity and remineralization potential. According to the results of the present study, bioactive cements and CPP-ACP exhibited bioactive properties after one week; the Biodentine cement was clearly more bioactive than others. Besides, demineralized dentin was remineralized by bioactive cements or bioactive amorphous cements (23).

In the FTIR analysis, the emergence of apatite and phosphate carbonate bonds with different peak intensities, using 1 wt.% and 2.5 wt.% ACP NPs and 1 wt.% and 2.5 wt.% CS NPs, confirmed the apatite formation and bioactivity; the peak intensity was relatively higher in ACP than in CS. Therefore, differences in composition can affect the amount and rate of apatite deposition. The hypothesis of this study, which assumed no significant difference in the bioactivity of the five bonding groups (0 wt.% (control group), 1 wt.%, and 2.5 wt.% CS and 1 wt.% and 2.5 wt.% ACP), was rejected.

In another study, Collins *et al*. confirmed that hydroxyapatite with various surfactants, synthesized by the emulsion method, showed the sharpest peak of phosphate in the apatite in the range of 1000-1100 cm-1, as shown in the FTIR diagram (24). Moreover, in a study by Shojaei *et al*., dental adhesives showed a phosphate peak in the range of 1000-1100 cm-1. Besides, the emergence of duplex peaks at wavelengths of approximately 1415 and 1451 cm-1 in the samples after immersion indicated carbonate impurities in the hydroxyapatite structure. Also, the peak at 3511 cm-1 corresponds to the bonding of hydroxyl groups in the crystalline structure of hydroxyapatite, as shown in the FTIR diagram (25).

In the present study, the XRD analysis also confirmed the formation of a nanocrystalline hydroxyapatite layer in FTIR after immersion in the SBF solution. In all groups, the main peak of the amorphous bioactive phase was in the 2θ range of 12-25°, as similarly reported in a study by Ravanbakhsh *et al*. (2θ=24.5°) in the XRD analysis (26). The enamel and natural mineral sediment structures were characterized in the three groups. In all three experimental groups, the remineralized enamel showed the same diffraction pattern compared to the normal enamel (at 43, 64.1, 39, and 36°), suggesting the presence of the same crystalline structure.

Additionally, peak diffraction at a 2θ angle of 33° seems possible, according to studies on the remineralization of primary caries on enamel, using an ACP carrier. The study by Shojaei *et al*. also confirmed this finding (5). The mentioned peaks and angles verified the formation of hydroxyapatite crystals, whereas the width of the peaks indicated the low crystallinity of these sediments, as similarly reported by Cao *et al*. (17). In this regard, Gabriel *et al*. studied bioactivity and biomimetic efficiency by incorporating phosphoprotein fillers into self-etch bands. In line with our study, demineralized dentin samples, prepared with a band containing calcium phosphate fillers and a primer containing polyacrylic acid or sodium triphosphate, showed a higher phosphate content (27).

According to the present results, there was no significant difference in the dentin bond strength between the groups of 2.5 wt.% ACP and 2.5 wt.% CS and the control group. Recent studies have shown that the use of bioactive materials, with a resin base etched on dentin (stored in body simulation fluids), can maintain the bond strength, while decreasing the nano-leakage and permeability at the resin-dentin interface (4).

Contrary to our study, Gabriel *et al*. reported that adding fillers, with or without primer surface preparation, could increase the tensile bond strength for six months. However, in the control group, with a primer and a band without a filler, a decrease in bond strength was observed after six months (27). In some studies, orthodontic adhesives containing ACP showed less bond strength than conventional adhesives; nonetheless, their clinical results were satisfactory (28). Moreover, bonding systems containing ACP have shown less flexural strength compared to conventional adhesives (29).

## Conclusions

According to the results of the present study, CS and ACP NPs can induce remineralization in the dentin. The formation of a hydroxyapatite layer after immersion in SBF solution was confirmed by the FTIR and XRD analyses. There was no significant difference in the average bond strength of the control group materials with the other one. Therefore, the addition of up to 2.5 wt.% CS or ACP NPs to the bond improved the remineralization potential and bioactivity, while it had no negative effects on the bond strength. Overall, the CS and ACP NPs are suitable candidates for improving the bio-functionalization of dentin bonds.
